# Case Report: Symptomatic Chronic Granulomatous Disease in the Newborn

**DOI:** 10.3389/fimmu.2021.663883

**Published:** 2021-03-29

**Authors:** Milica Miladinovic, Boris Wittekindt, Sebastian Fischer, Elise Gradhand, Steffen Kunzmann, Stefanie Y. Zimmermann, Shahrzad Bakhtiar, Thomas Klingebiel, Rolf Schlösser, Thomas Lehrnbecher

**Affiliations:** ^1^ Hospital for Children and Adolescents, University Hospital of Frankfurt, Frankfurt, Germany; ^2^ Institute of Pathology, University Hospital of Frankfurt, Frankfurt, Germany; ^3^ Department of Diagnostic and Interventional Radiology, University Hospital of Frankfurt, Frankfurt, Germany; ^4^ Clinic of Neonatology and Pediatric Intensive Care, Bürgerhospital, Frankfurt, Germany

**Keywords:** chronic granulomatous disease, neonate, early onset, symptoms, outcome

## Abstract

Chronic granulomatous disease (CGD) is a primary immunodeficiency, which is diagnosed in most patients between one and three years of age. Here we report on a boy who presented at birth with extensive skin lesions and lymphadenopathy which were caused by CGD. An analysis of the literature revealed 24 patients with CGD who became symptomatic during the first six weeks of life. Although pulmonary complications and skin lesions due to infection were the leading symptoms, clinical features were extremely heterogenous. As follow-up was not well specified in most patients, the long-term prognosis of children with very early onset of CGD remains unknown.

## Introduction

Chronic granulomatous disease (CGD) is a rare primary immunodeficiency which occurs with a frequency of approximately 1:200.000 in the United States and Europe and is characterized by an increased susceptibility to bacterial and fungal infections (1–3). The disease is caused by a defect of the NADPH oxidase complex and most of the mutations are located in the genes gp91phox, p47phox, p22phox, p67phox or p40phox (4, 5). In affected families, patients may be diagnosed prior to any signs of the disease, even prenatally. However, the vast majority of patients with CGD is diagnosed between one and three years of age when they become clinically symptomatic with recurrent and severe infectious complications, mostly affecting the lung (79%), lymph nodes (53%), liver (27%) and skin (42%) (3). Typical pathogens include *Staphylococcus aureus*, *Burkholderia cepacia*, *Serratia marcenscens, Nocardia* spp, and *Aspergillus* spp, in particular *A. fumigatus* and *A. nidulans* (2–4, 6). Notably, CGD can also present in unusual forms such as gastrointestinal mucormycosis, cardiac empyema or phlebitis, which makes early diagnosis difficult, in particular in the neonatal period (3, 7). A milder phenotype of the disease has been associated with later diagnosis, but also with longer survival (2).

Here we describe the unusual case of a neonate with CGD who presented with extensive skin lesions and lymphadenopathy at birth, which prompted us to review and analyze the current literature for patients with an extremely early onset of CGD.

## Case Presentation

**Figure 1:**
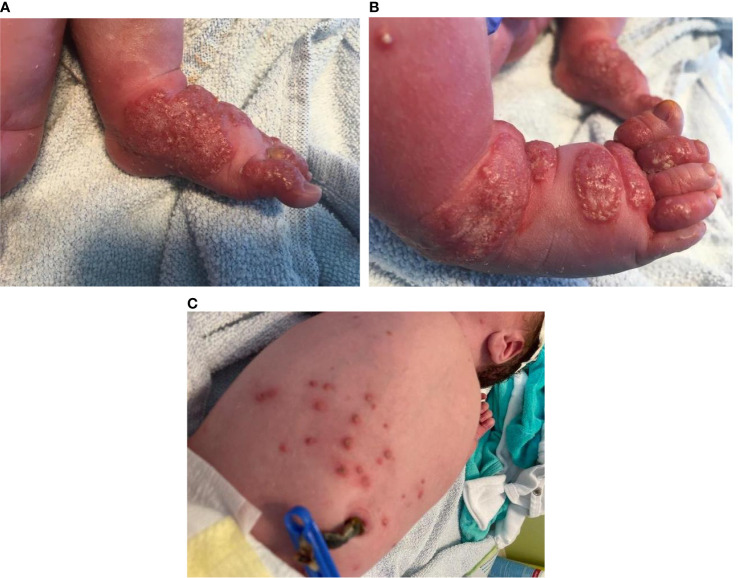
**(A**, **B)** Papulo-pustular lesions on an erythematous base on the feet. **(C)** Scattered papules with central vesicles on the body in a newborn with chronic granulomatous disease.

After uneventful pregnancy, a full-term neonate presented at birth with extensive papulo-pustular lesions on both hands and feet and scattered papules with central vesicles on the body (**Figure 1**). The boy was in good clinical condition, no other abnormality was seen. The father and the 6-year-old half-brother were healthy, whereas the mother was diagnosed at the age of 20 years with Crohn´s disease and was currently under therapy with the monoclonal antibody vedolizumab.

**Figure 2:**
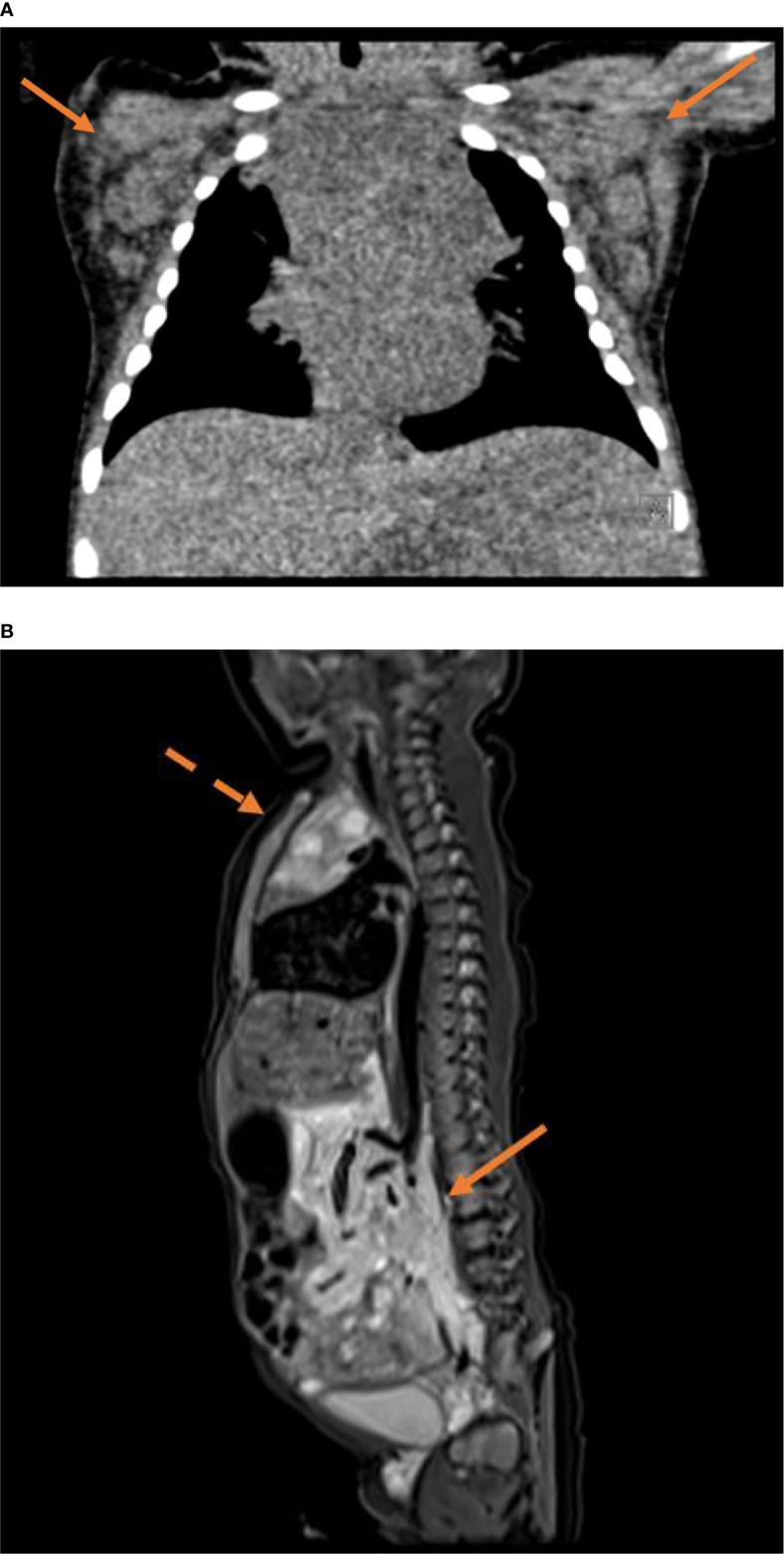
**(A)** Noncontrast CT (80kV, 48mAs, FOV 170x130mm) with coronal reconstruction using a soft tissue kernel shows a distinct bilateral axillary lymphadenopathy (arrows) and a prominent inhomogeneous thymus. **(B)** Sagittal T2 STIR sequence of a whole body MRI (TE 33ms, TR 3800ms, FOV 300x300mm, matrix 256x256px) shows bulky hyperintense soft tissue masses surrounding the aortocaval and mesenteric vasculature (solid arrow). The enlarged thymus features multiple jagged-edged cystic lesions (dotted arrow).

Laboratory tests revealed leukocytosis (25,560/µl, upper limit 16,200/µl), a high absolute number of eosinophils (up to 5340/µl, upper limit 950/µl) and an elevated C-reactive protein (initial evaluation 7.7 mg/dl, maximum 21.77 mg/dl; normal range <0.4mg/dl). Immunologic parameters including lymphocyte subsets and immunoglobulins were within normal range. Tumor markers including alpha-fetoprotein and ß-HCG were negative, and a chromosomal analysis did not reveal abnormalities. The blood level of vedolizumab six weeks after birth was with <4.0 µg/ml below the detection limit. Despite cultures of blood and skin lesions remained negative, antibiotic therapy was initiated, but showed no significant effect on leukocytes and C-reactive protein. Imaging studies revealed an enlarged thymus with multiple jagged-edged cysts (**Figure 2**), and axillary lymph nodes as well as those located along the lateral thoracic wall, parailiacal and inguinal were also increased in size with a maximum diameter of 1.8 cm. Magnetic resonance imaging showed bulky soft tissue masses surrounding the abdominal aorta and its branches from the coeliac trunk to the external iliac artery (**Figure 2**).

**Figure 3:**
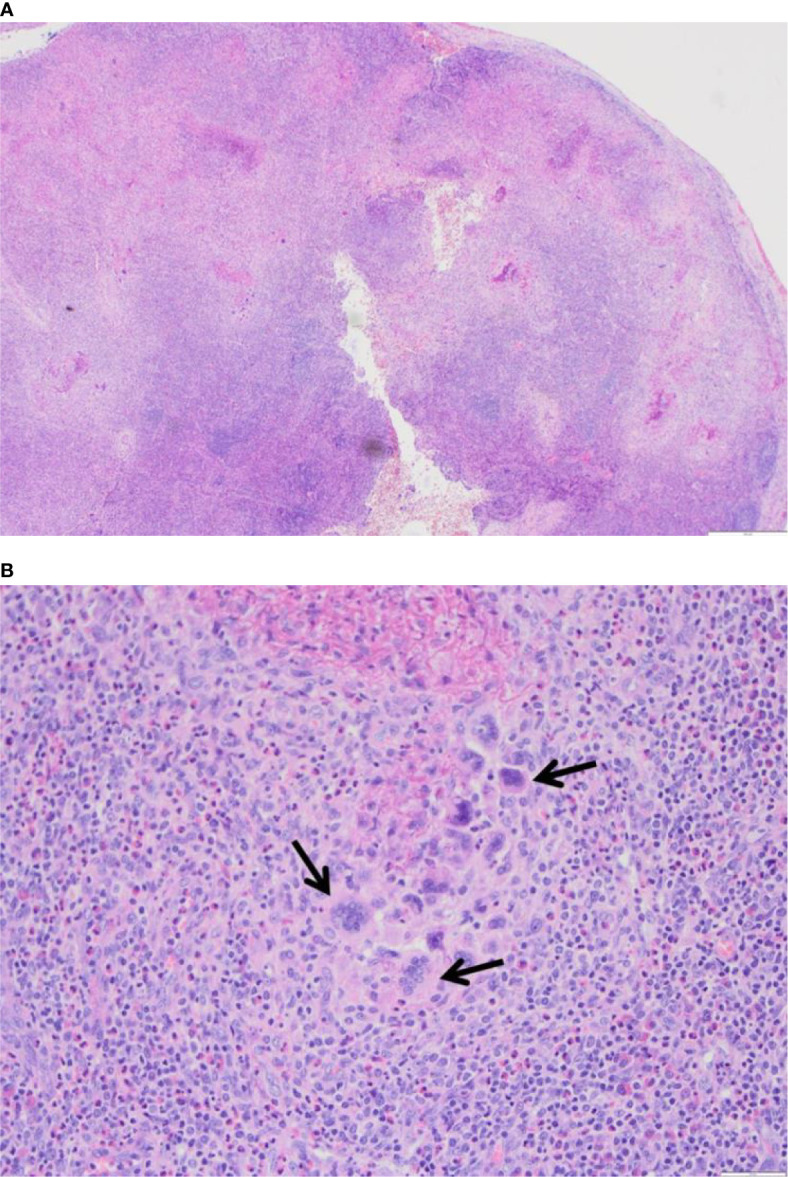
**(A)** H+E, 4x, Lymph node with severely disturbed architecture by a diffuse necrotizing and granulomatous inflammation. **(B)** H+E, 4x, Lymph node, close-up of the granulomatous inflammation with abundant multinucleated giant cells (see arrows) in a background of neutrophils and eosinophils.

A biopsy of the skin and a lymph node revealed granulomatous inflammation with eosinophilic infiltrates. The granuloma showed a central collection of amorphous necrotic but not caseating material of fragmented fibres and prominent multi-nucleated giant cells. No atypical mycobacteria were detected, and CD1a and langerin expression were absent (**Figure 3**).

Additional Immunologic investigations revealed a pathologic function of the NADPH-oxidase (DHR assay 1.40%, normal >98%), and a mutation within the hemizygous CYBB gene (c.742dupA, which results in a premature stop of translation) confirmed the diagnosis for X-linked CGD. This mutation was not found in the half-brother, genetic testing of the mother is planned. Several weeks later, the boy developed pulmonary granuloma due to probable invasive aspergillosis and/or auto-inflammation. Allogeneic hematopoietic stem cell transplantation was performed at the age of 4 months, without major complication during the first three weeks post-transplant.

## Extremely Early Onset of CGD—Review of the Literature

References without language restriction were retrieved from MEDLINE (including MEDLINE In-Process) database up to February 28, 2021). The search included terms such as neonate, newborn, CGD, and chronic granulomatous disease. Retrieved publications were manually screened for additional references. Patients were included in the analysis if they developed symptoms compatible with CGD within the first six weeks of life, and CGD was diagnosed either by functional tests of neutrophils (including nitroblue tetrazolium test (NBT), dihydrorhodamine (DHR) assay or chemiluminescence test) and/or by genetic analysis. Patients without clinical symptoms detected by screening as family members of CGD-index candidates were not included in the analysis.

The search identified 24 patients, eight girls and sixteen boys (**Table 1**). Four of the patients were symptomatic already at birth. The diagnosis of CGD was made at an average age of 8 months (range, 1 month to 2 years 8 months), and a mutational analysis was reported in 18 out of the 24 patients. Eleven patients had a mutation in the gp91phox gene, whereas a mutation p67phox was found in three patients and in the p22phox and p47phox gene in two patients each (**Table 1**). The most common symptoms were respiratory problems (n=13; pulmonary nodules detected by imaging studies in 12 patients), skin lesions such as papules or abscess (11/24 patients) and fever (12/24 patients). Less often, lymphadenopathy (5/24 patients) or gastrointestinal symptoms (5/24 patients) were seen. Most patients had elevated inflammatory parameters (17/24 patients).

**Table 1:** Summary of patients reported in the literature with onset of chronic granulomatous disease (CGD) within the first six weeks of life.

Sex (reference)	Presenting symptoms	Imaging studies	Other relevant findings	Pathogen isolated	Diagnosis of CGD	Therapy and outcome
Boy (reported patient)	Papulo-pustular lesions, lymph-adenopathy	Enlarged thymus, lymphadenopathy (CT)	Skin and lymph node histopathology: granuloma, eosinophils	n.a.	DHR assay 1.4%, (gp91phox mutated: c.742dupA)	Allogeneic HSCT at 4 months of age
Boy (7)	Pneumonia, pustular rash, fever, diarrhea sepsis	Abdominal tumor, lymphadenopathy (CT)	Histopathology: abscess, granulomatous reaction, giant cells	*S.aureus*, *Rhizopus microsporus*	NBT negative (no genetic analysis reported)	Antifungal prophylaxis, no HSCT. At 15 years of age no major complication
Boy (8)	Pustular rash, fever	Multiple lesions in liver and lung (CT)	Lung and liver histopathology: neutrophilic abscesses	*Serratia marcescens, Aspergillus spp*	NBT 0% (gp91phox mutated) X-linked cytochrome b558	Resolution of all lesions with antibiotic therapy
Boy (9)	Pneumonia, fever	Multiple pulmonary nodules (CT)	Lung histopathology: Histiocytic granuloma, giant cells, eosinophils	*E. coli*, *A. fumigatus*	Chemiluminescence pathologic (gp91phox mutated) X-linked cytochrome b558	n.a.
Girl (10)	Pneumonia, sepsis, lymph-adenopathy, gastroenteritis	Pulmonary nodules (CT)	Galactomannan in BAL^5^	*Enterobacter aerogenes*	NBT negative (no genetic analysis reported)	HSCT at 1 year of age
Girl (11)	Erythematous pustules and nodules	Multifocal osteomyelitis (radiograph)	n.a.	*Serratia marcescens*	Absent DHR response (autosomal recessive, p22phox mutated: nonsense mutation (261 C>A)	No clinical symptoms with therapy
Boy (12)	Lethargy, fever	Pulmonary infiltrates (CT)	Lung histopathology: necrotizing infection with focal microabscesses	*Nocardiaactinomyces*, *N. asteroides*	NBT negative (no genetic analysis reported)	n.a.
Boy (13)	Pustular rash, dactylitis	Multifocal osteomyelitis (radiograph)	Skin histopathology: necrotizing, granulomatous	*S. aureus*	NBT and DHR pathologic (no genetic analysis reported)	n.a.
Girl (14)	Respiratory problems, diarrhea	Bilateral pneumonia (X-ray)	n.a.	*Aspergillus spp*	NBT 0% (autosomal recessive, p47phox mutated)	Polyarthritis at the age of 4 years
Girl (15)	Respiratory problems, subcutaneous granuloma	Pulmonary infiltrates (CT)	n.a.	*A. fumigatus*	Superoxide generation pathologic (autosomal recessive, p67phox mutated)	Allogeneic HSCT at the age of 9 months, then no clinical problems
Girl (16)	Respiratory problems, fever	Pulmonary infiltrates (CT)	Lung histopathology: granuloma, giant cells	n.a.	No DHR response (autosomal recessive p67phox mutated: Glu260X/Arg395Trp)	Allogeneic HSCT in the first year of life, Healthy at 9 years of age
Boy (17)	Fever, perineal ulcerations	n.a.	n.a.	*Klebsiella oxytoca, Enterococcus faecium*	No DHR response (gp91phox mutated)	n.a.
Boy (18)	Swelling of the finger	Osteomyelitis (radiograph)	n.a.	*Serratia marcenscens*	Not reported (gp91phox mutated)	Allogeneic HSCT at the age of 2 years. Healthy at 7 years of age
Boy (19)	Respiratory problems, fever	Pulmonary infiltrates (CT)	Histopathology: microabscess formation with granulomas	*Aspergillus spp*	NBT negative (gp91phox mutated)	n.a.
Girl (20)	Respiratory problems, fever, lymphadenitis, gluteal abscess	Pulmonary infiltrates and cavitation (CT)	n.a.	*S. aureus*	NBT 5% (no genetic analysis reported)	n.a.
Girl (21)	Fever	Pulmonary infiltrates, mediastinal mass (CT)	Lung histopathology: inflammatory cells	*A. fumigatus*	NBT negative (autosomal recessive, p22phox mutated)	n.a.
Girl (22)	Retropharyngeal abscess, lymphadenopathy, gastroenteritis	n.a.	n.a.	*Campylobacter spp, Serratia marcesens, Klebsiella oxytoca*	No superoxide producing granulocytes (gp91phox mutated: heterozygous mutation in exon 5 (c.469C>T)	Infection free for 6 months
Boy (23)	Pustular rash, fever, lymph-adenopathy	Pulmonary infiltrates (CT), lesions in liver and spleen (US)	n.a.	n.a.	NBT negative, no DHR response (no genetic analysis reported)	n.a. (HSCT planned)
Boy (24)	Liver and skin abscesses, sepsis	n.a.	n.a.	*Enterobacter* spp.*, Klebsiella spp*	n.a. (gp91phox mutated)	Abscesses decreased in size
Boy (24)	Skin abscess lymphadenopathy	n.a.	n.a.	*Serratia* spp. *M. bovis*	n.a. (gp91phox mutated: c.674+5G>A)	alive
Boy (24)	Skin abscess	n.a.	n.a.	*Serratia marcescens*	n.a. (autosomal recessive, NCF1: c.75_76delGT)	n.a.
Boy (25)	Pneumonia, sepsis	n.a.	n.a.	n.a.	n.a. (gp91phox mutated)	n.a.
Boy (25)	Pneumonia	n.a.	n.a.	n.a.	n.a. (gp91phox mutated)	n.a.
Boy (26)	Fever, cough	Pulmonary infiltrates (CT)	n.a.	*A. fumigatus*	n.a. (autosomal recessive, p67phox mutated)	Frequent infections, progressive pulmonary lesions
Boy (27)	Fever, abdominal distension, pallor	Hepatosplenomegaly, ascites, splenic micro- abscesses (US)	Hemophagocytosis of bone marrow, pathologic coagulation	n.a.	NBT negative, no DHR response (gp91phox mutated: c.1429G>A, p.Trp443X)	Death during hospital stay despite antimicrobial therapy

CT, computerized tomography; US, ultrasound; NBT, nitroblue tetrazolium test; DHR, dihydrorhodamine assay; HSCT, hematopoietic stem cell transplantation; n.a., not available.

In 19 out of the 24 patients, a pathogen was isolated (bacteria in 11 patients, a fungus in 5 patients, both bacterial and fungal pathogens in 3 patients) (**Table 2**). The most frequent pathogens were *Aspergillus* spp (7/24 patients). Bacterial infections were mainly caused by *Gram*- negative bacteria, mostly by *Serratia* spp. (+6/24 patients).

**Table 2:** Pathogens isolated in 19 patients with onset of chronic granulomatous disease within the first six weeks of life.

Bacteria	Patients
*Gram*-positive	
*Staphylococcus aureus*	3
	
*Enterococcus faecium*	1
*Nocardia spp*	2
*Mycobacterium bovium*	1
	
*Gram*-negative	
*Serratia* spp	6*
*E. coli*	1
*Klebsiella* spp	2
*Enterobacter* spp	2
*Campylobacter* spp	1
	
Fungi	
*Aspergillus* spp	7**
*Rhizopus microsporus*	1

*5 patients with Serratia marcescens.

**4 patients with A. fumigatus; 3 additional patients suffered from probable invasive aspergillosis (galactomannan positive, pulmonary infiltrates).

## Discussion

Chronic granulomatous disease is a rare primary immunodeficiency which is caused by a defect of the NADPH oxidase (1). Due to the impairment of the phagocytic function, patients have a high risk of bacterial and fungal infections (1). The majority of patients become symptomatic in childhood, but rarely within the first weeks of life (2, 3, 6). As we saw a boy with extended skin lesions caused by CGD already at birth, we thought to review the current literature for patients with extremely early onset of CGD. In total, we identified 24 patients who developed symptoms of CGD within the first six weeks of life. As in our analysis, corresponding studies in older children and adults report on a slight preponderance of boys (2, 3). Corroborating previous reports of older patients, the majority of symptomatic neonates suffered from the X-linked form of CGD, as did our patient (2, 3). Similarly, respiratory problems, skin lesions and fever were the most common initial symptoms of CGD in patients with very early onset of disease, which is comparable to older patients (2, 3). Abscesses are typical skin lesions adults with CGD, whereas in younger children, the lesions are extremely variable (2, 3). It has been reported that life-threatening hemophagocytic lymphohistiocytosis (HLH) presenting with a number of signs and symptoms including persistent fever, hepatosplenomegaly, lymphadenopathy, and low counts of red blood cells, white blood cells and platelets may be the first manifestation of CGD, which is not surprising as any infection can trigger secondary HLH (27–29). In our analysis, erythematous or vesiculo-pustular lesions were found in five patients, and six of them presented with skin abscesses. None of the neonates suffered from extended papulo-pustular and erythematous skin lesions comparable to our patient, which were recently described in two infants of 4 and 9 months of age, respectively (30). Interestingly, these patients had similar histopathologic findings and the same CYBB mutation as our patient (30). Eosinophilic inflammation and an elevated number of eosinophils, as observed in our patient, has been described in patients with X-linked CGD (31). This fact might be due to a compensatory mechanism for the neutrophil defect, as eosinophils may be able to produce gp91phox due to differential regulation of expression of this protein (32). In addition, eosinophilic major basic protein has been shown to activate neutrophils by increasing NADPH oxidase activity, and therefore, one can speculated whether the elevated number of eosinophils are a response to the deficient NADPH oxidase system (31).

In contrast to our newborn patient, a bacterial and/or fungal pathogen was isolated in most of the neonatal patients reported in the literature, with *Staphylococcus aureus* and *Serratia* spp as the predominant bacterial pathogens. This observation corroborates the findings in adults patients with CGD that *Staphylococcus aureus* is the most frequent pathogen causing abscesses and pulmonary infiltrates and that *Serratia* spp is frequently isolated in subcutaneous abscesses and osteomyelitis (3, 33). Almost half of the patients of our analysis suffered from probable or proven invasive fungal infection, mostly caused by *Aspergillus* spp. A large US registry including 386 children and adults with CGD reported that over time, invasive aspergillosis was diagnosed in 41% of the patients, and that *Aspergillus* spp was responsible in 35% of all infections with lethal outcome (3). Interestingly, in none of the patients in our analysis, *A. nidulans* was isolated, which is the second most encountered mold in CGD patients (34). Due to highly variable symptoms, early diagnosis of CGD in the very young is difficult, which explains the fact that in neonates with CGD, the diagnosis of CGD was made at an average age of 8 months. Newborn screening tests may help to diagnose and treat patients with CGD early, and recently, a robust novel method was reported which allowed the identification of neonates with various primary immunodeficiencies including X-linked CGD (35). Notably, in our patient, the mother suffered from Crohn’s disease, which has been described in female carriers (36), and therefore, genetic testing of the mother is being planned. The fact that the mother was treated with vedolizumab, a humanized monoclonal antibody to α4β7 integrin detected on a specific subpopulation of memory T-lymphocytes for down-regulation of inflammatory processes in the gastrointestinal tract did not explain the symptoms of our patient according to the information given in the literature (37–39). In addition, studies show that vedolizumab levels assessed in cord blood are lower than maternal levels and clear rapidly, with blood levels below the detection limit at 6 weeks after birth (40).

It is important to note that the histopathologic findings of cutaneous granulomas may be indicative of primary immunodeficiency (41), but also feature associations with Crohn´s disease, sarcoidosis, Langerhanscell histiocytosis or tuberculosis (42). Comparable histopathological findings were reported in five out of the 24 patients of our analysis, but in four of them an additional pathogen was detected. Eosinophils were not abundant.

Standard of care of patients with CGD consists of prophylaxis and treatment with antibacterial and antifungals agents (43, 44). However, it is important to note that in the neonatal age group, none of the commonly used broad-spectrum triazoles such as itraconazole or posaconazole is approved nor an adequate dosage has been established (45–47). Similarly, the benefit of interferon-γ, which significantly reduced the incidence of serious infections in a double-blind placebo-controlled study enrolling 128 patients with CGD (median age, 15 years), is not clear at all in the very young age group (48). To date, cure is only achieved by hematopoietic stem cell transplantation (HSCT), which results in a survival rate of more than 80% (43, 49). In our analysis, limited data regarding follow-up was provided for only ten patients. Unfortunately, the information is insufficient for a solid conclusion whether patients with an extremely early onset of CGD have a worse outcome compared to those with a later onset, and is clearly a limitation of this analysis.

Our data demonstrate that unspecific skin lesions and pulmonary symptoms during the first weeks of life may indicate very early onset of CGD. To date, it is unclear whether these patients have a worse prognosis than those which a later onset of the disease.

## Conclusion

Chronic granulomatous disease is a life-threatening genetic immunodeficiency, which is diagnosed in the majority of patients between one and three years of age when they become clinically symptomatic. Our patient presented already at birth with unusual skin lesions and lymphadenopathy. A review of the literature revealed only 24 patients who presented with symptomatic CGD within the first weeks of life. Clinical features were extremely heterogenous. As follow-up data of these patients are limited, it remains unclear whether patients with an extremely early onset of CGD have a worse prognosis than those with a later onset of disease.

## Data Availability Statement

The original contributions presented in the study are included in the article/supplementary material. Further inquiries can be directed to the corresponding author.

## Ethics Statement

Written informed consent was obtained from the minor(s)’ legal guardian/next of kin for the publication of any potentially identifiable images or data included in this article.

## Author Contributions

MM and TL performed the literature research, analyzed data, and drafted the manuscript. BW, SK, SYZ, SB, TK, and RS analyzed clinical data. SF analyzed radiological data. EG analyzed pathological data. All authors contributed to the article and approved the submitted version.

## Conflict of Interest

The authors declare that the research was conducted in the absence of any commercial or financial relationships that could be construed as a potential conflict of interest.
